# Correction: Zheng et al. Glycolysis-Related SLC2A1 Is a Potential Pan-Cancer Biomarker for Prognosis and Immunotherapy. *Cancers* 2022, *14*, 5344

**DOI:** 10.3390/cancers15030586

**Published:** 2023-01-18

**Authors:** Haosheng Zheng, Guojie Long, Yuzhen Zheng, Xingping Yang, Weijie Cai, Shiyun He, Xianyu Qin, Hongying Liao

**Affiliations:** 1 Department of Thoracic Surgery, Thoracic Cancer Center, The Sixth Affiliated Hospital, Sun Yat-sen University, Guangzhou 510655, China; 2 Guangdong Research Institute of Gastroenterology, The Sixth Affiliated Hospital, Sun Yat-sen University, Guangzhou 510655, China; 3 Department of Pancreatic Hepatobiliary Surgery, The Sixth Affiliated Hospital of Sun Yat-sen University, Guangzhou 510655, China

The authors wish to make the following corrections to this paper [1]:

In the published version, there were mistakes in “3.9. Immune Cell Infiltration Analysis of SLC2A1” of page 14 and Figure 10. 

On page 14, in the Section “3.9. Immune Cell Infiltration Analysis of SLC2A1”.

The sentence “SLC2A1 expression is negatively correlated with CD8+ T cells in 12 types of cancers (STES, TGCT, ESCA, LUSC, SKCM, LUAD, BLCA, HNSC, CESC, LAMLC, THYM, and GBM), but positively with CD8+ T cells in 5 types of cancers (PRAD, KIPAN, KIRP, CHOL, and LIHC) (Figure 13A).” should be changed to “SLC2A1 expression is negatively correlated with CD8+ T cells in 9 types of cancers (LUSC, TGCT, HNSC, CESC, LUAD, LAML, SKCM, THYM, and GBM), but positively with CD8+ T cells in 4 types of cancers (PRAD, KIPAN, UVM, and CHOL) (Figure 13A).”

The sentence “SLC2A1 expression is negatively correlated with CD8+ T cells in 12 types of cancers (LUSC, TGCT, THYM, HNSC, BRCA, SKCM, STES, GBMLGG, GBM, PAAD, ALL, and ESCA), but positively correlated with CD8+ T cells in 5 types of cancers (KIPAN, LIHC, LAML, PCPG, and CHOL) (Figure 13B).” should be changed to “SLC2A1 expression is negatively correlated with CD8+ T cells in 11 types of cancers (LUSC, TGCT, THYM, HNSC, BRCA, SKCM, STES, GBMLGG, GBM, PAAD, and ESCA), but positively with CD8+ T cells in 5 types of cancers (KIPAN, LIHC, LAML, PCPG, and CHOL) (Figure 13B).”

The gene symbols of Figure 10 were not correct. The corrected Figure 10 appears below. 

The authors emphasize that the mistakes were entirely due to human error and oversight. The corrections do not affect the main scientific results and the final conclusions of this manuscript. The authors would like to apologize for any inconvenience caused. The original article has been updated.

## Figures and Tables

**Figure 10:**
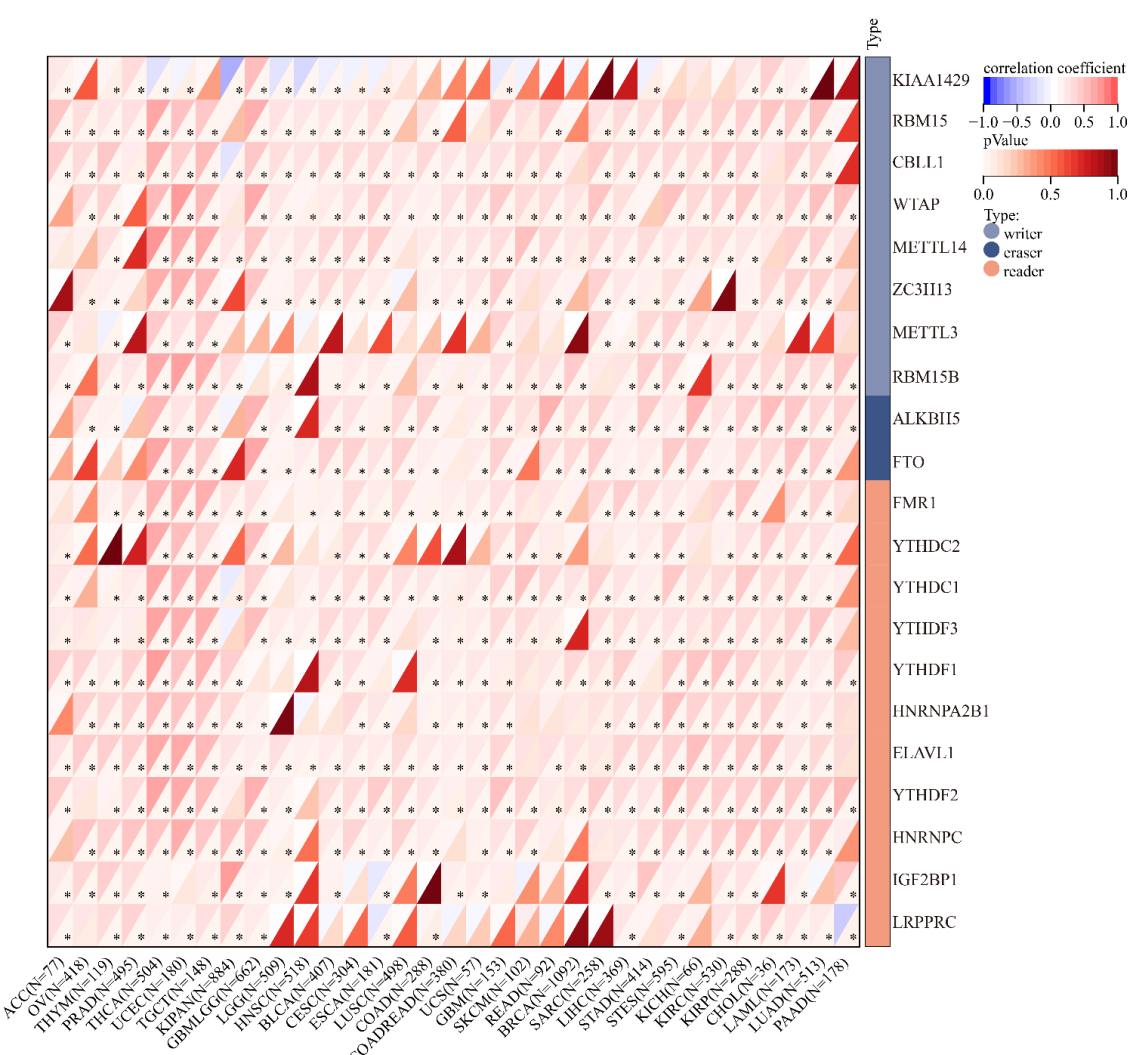
Relationship between SLC2A1 expression and RNA m6A-methylation-related genes in pan-cancer (*, *p* < 0.05).
