# Supplementary material for: Glycolysis-Related SLC2A1 Is a Potential Pan-Cancer Biomarker for Prognosis and Immunotherapy
Source: Cancers (Basel). 2022 Oct 29;14(21):5344. doi: 10.3390/cancers14215344 (PMC9657346; doi:10.3390/cancers14215344)
Supplement: Supplementary file 1 [file cancers-14-05344-s001.zip › cancers-1966936-Supplementary Materials.pdf]

Supplementary Materials: Glycolysis-Related SLC2A1 Is a Potential Pan-Cancer Biomarker for Prognosis and Immunotherapy

Haosheng Zheng, Guojie Long , Xianyu Qin, Yuzhen Zheng , Xingping Yang , Weijie Cai , Shiyun He and Hongying Liao

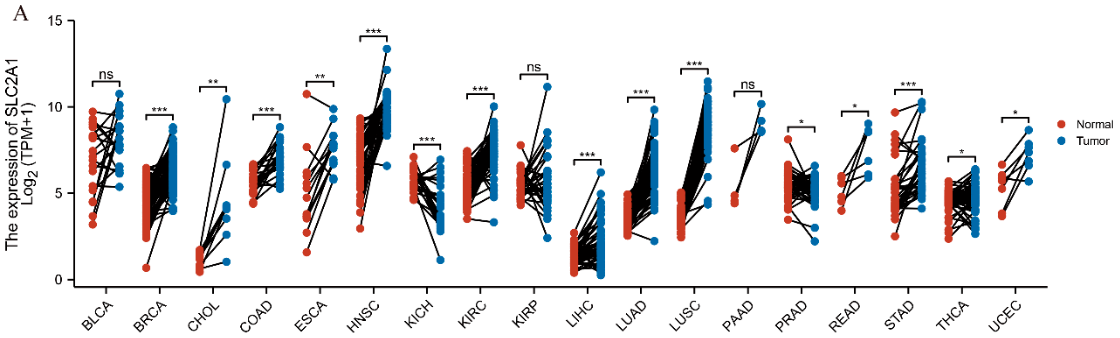

Figure S1. SLC2A1 expression in TCGA cancers and adjacent normal tissues(ns, p ≥ 0.05; *, p < 0.05; **, p < 0.01; ***, p < 0.001)

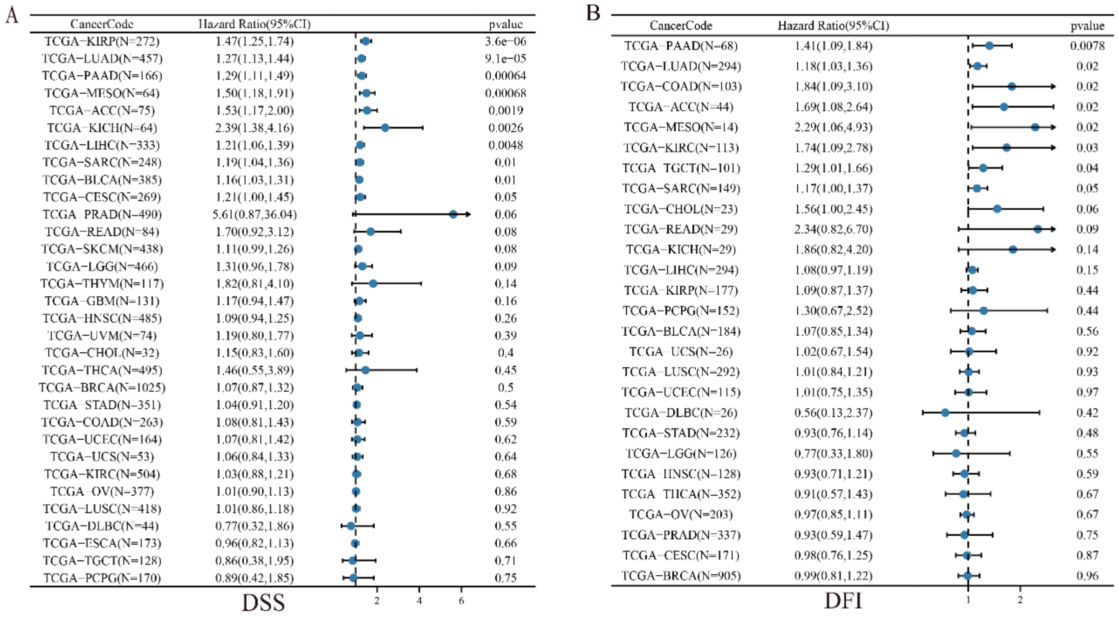

Figure S2. Univariate Cox regression analysis of SLC2A1. Forest map shows the univariate Cox regression results of SLC2A1 for DSS (A) and DFI (B) in pan-cancer

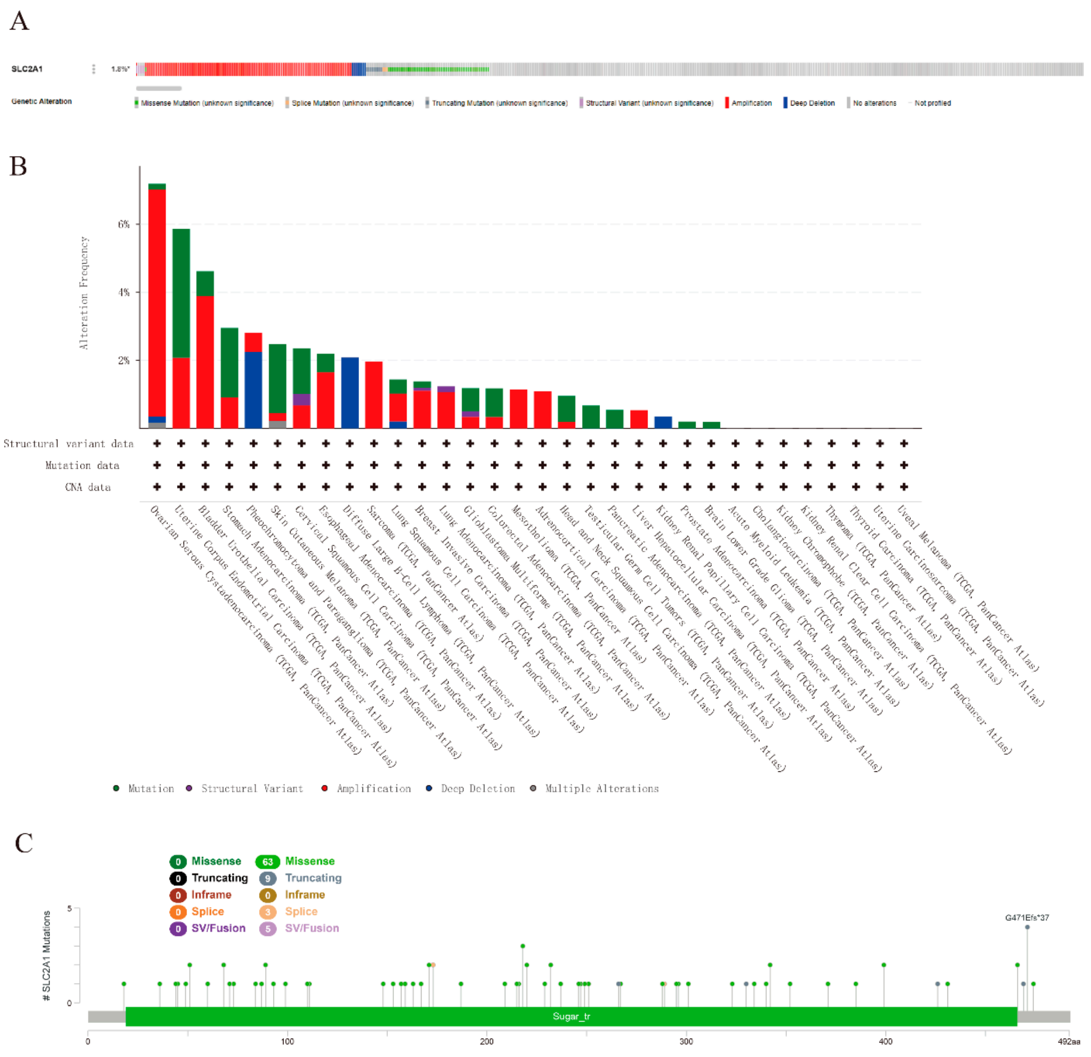

Figure S3. SLC2A1 genetic alterations in pan-cancer analyzed using cBioPortal database. OncoPrint of SLC2A1 genetic alterations in pan-cancer (different colors mean different types of genetic alterations) (A); SLC2A1 alteration types and frequency of pan-cancer (B); mutation types and sites of SLC2A1 gene (C);

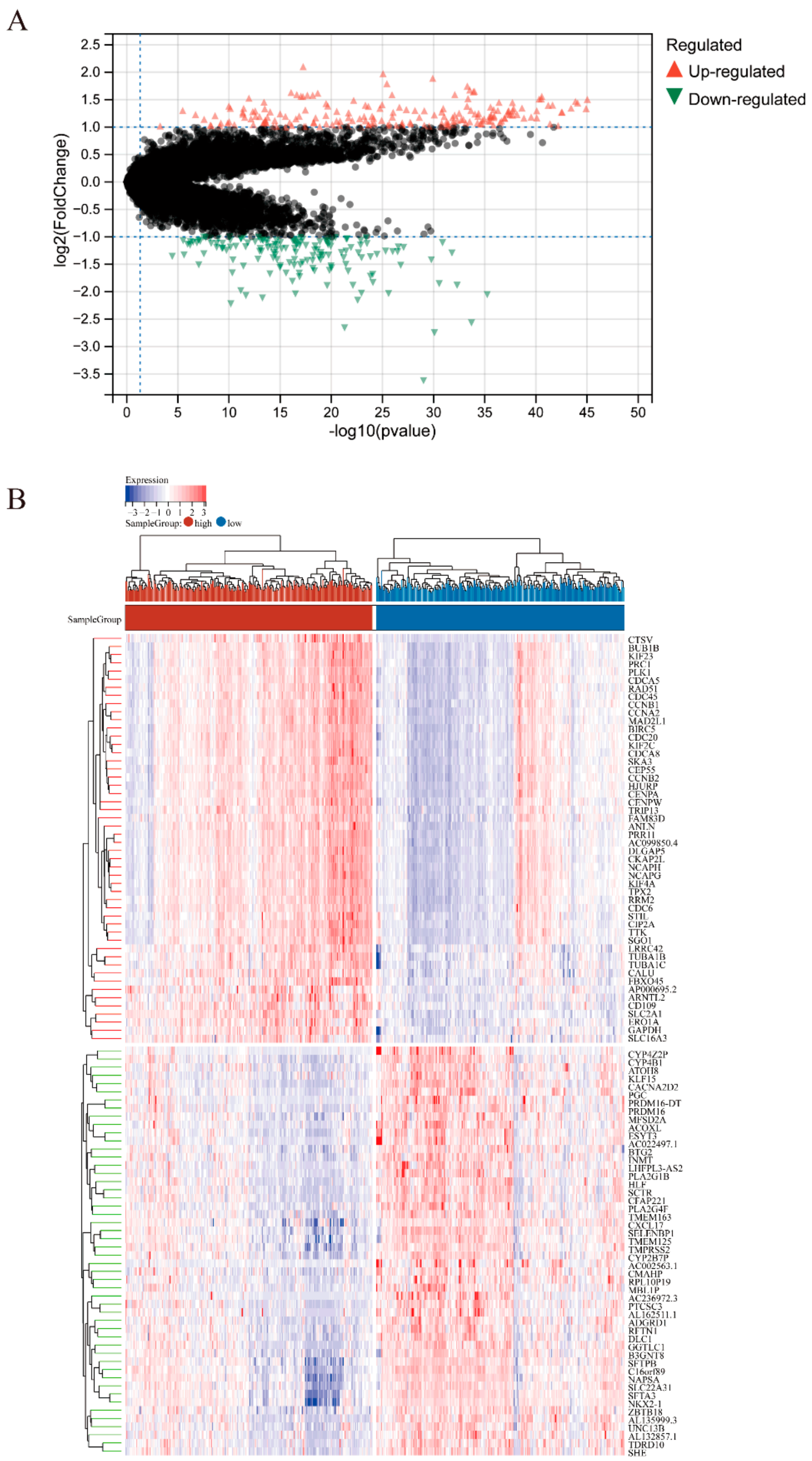

Figure S4. Identification of DEGs related to SLC2A1. Volcano plot depicts the 346 DEGs (|log2FC| > 1; FDR < 0.05) in TCGA LUAD group of high vs. low expression of SLC2A1 (A); heatmap shows top 50 upregulated and downregulated DEGs (B)

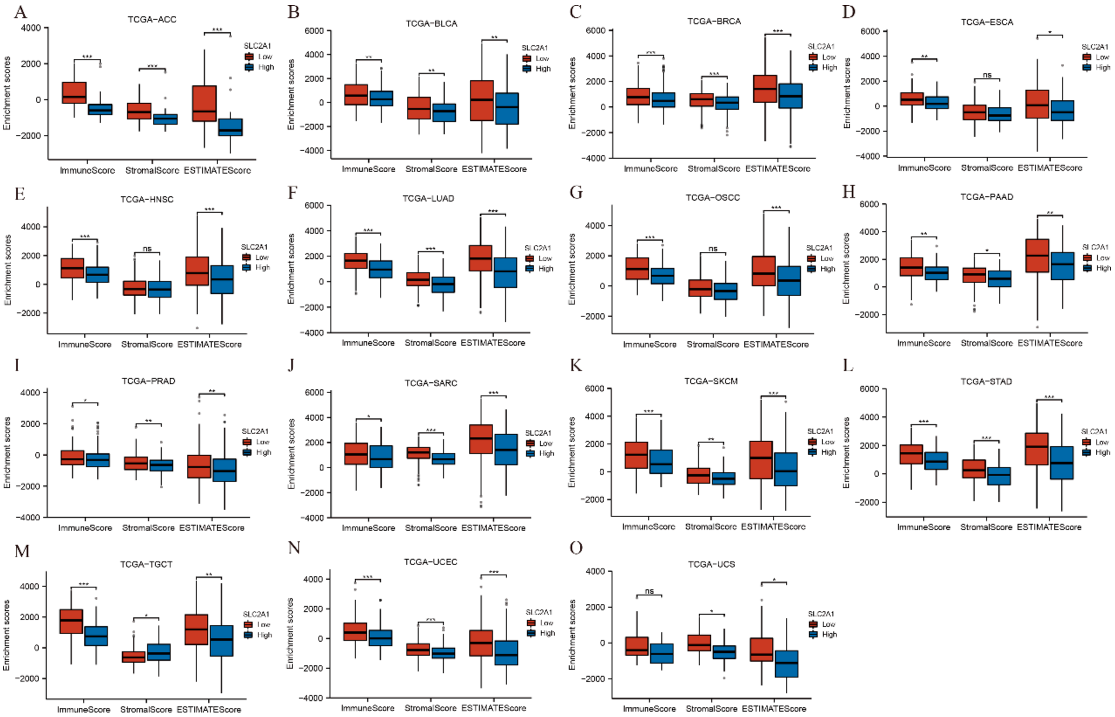

Figure S5. Differences in TME between high-SLC2A1 and low-SLC2A1 were evaluated by ESTIMATEScore algorithm(ns, $p \geq 0.05$; *, $p < 0.05$; **, $p < 0.01$; ***, $p < 0.001$)

Table S1. Abbreviations of all cancer types

Abbreviation	Full name
TCGA-ACC	Adrenocortical carcinoma
TCGA-BLCA	Bladder Urothelial Carcinoma
TCGA-BRCA	Breast invasive carcinoma
TCGA-CESC	Cervical squamous cell carcinoma and endocervical adenocarcinoma
TCGA-CHOL	Cholangiocarcinoma
TCGA-COAD	Colon adenocarcinoma
TCGA-COADREAD	TCGA-COAD + TCGA- READ
TCGA-DLBC	Lymphoid Neoplasm Diffuse Large B-cell Lymphoma
TCGA-ESCA	Esophageal carcinoma
TCGA-GBM	Glioblastoma multiforme
TCGA-GBMLGG	TCGA-GBM+ TCGA-LGG
TCGA-HNSC	Head and Neck squamous cell carcinoma
TCGA-KICH	Kidney Chromophobe
TCGA-KIPAN	Pan-kidney cohort (KICH+KIRC+KIRP)
TCGA-KIRC	Kidney renal clear cell carcinoma
TCGA-KIRP	Kidney renal papillary cell carcinoma
TCGA-LAML	Acute Myeloid Leukemia
TCGA-LGG	Brain Lower Grade Glioma
TCGA-LIHC	Liver hepatocellular carcinoma
TCGA-LUAD	Lung adenocarcinoma
TCGA-LUSC	Lung squamous cell carcinoma
TCGA-MESO	Mesothelioma
TCGA-OV	Ovarian serous cystadenocarcinoma
TCGA-PAAD	Pancreatic adenocarcinoma
TCGA-PCPG	Pheochromocytoma and Paraganglioma
TCGA-PRAD	Prostate adenocarcinoma
TCGA-READ	Rectum adenocarcinoma
TCGA-SARC	Sarcoma
TCGA-STAD	Stomach adenocarcinoma
TCGA-SKCM	Skin Cutaneous Melanoma
TCGA-STES	TCGA-STAD + TCGA-ESCA
TCGA-TGCT	Testicular Germ Cell Tumors
TCGA-THCA	Thyroid carcinoma
TCGA-THYM	Thymoma
TCGA-UCEC	Uterine Corpus Endometrial Carcinoma
TCGA-UCS	Uterine Carcinosarcoma
TCGA-UVM	Uveal Melanoma

Table S2. Basic information of GEO datasets in the study

Dataset	Cancer Type	Sample Type	
		Tumor	Normal
GSE2088	LUSC	38	30
GSE13507	BLCA	188	68
GSE10927	ACC	33	10
GSE39001	CESC	62	17
GSE26566	CHOL	100	36
GSE18520	OV	53	10
GSE53757	KIRC	72	72
GSE62452	PAAD	65	65
GSE87211	READ	203	160
GSE15605	SKCM	58	16
GSE33630	THCA	59	46
GSE3218	TGCT	101	6
GSE17025	UCEC	91	12
GSE47861	BRCA	76	85
GSE68468	COAD	106	195
GSE53625	ESCA	179	179
GSE13601	HNSC	32	26
GSE57927	LIHC	39	39
GSE75037	LUAD	166	166
GSE26899	STAD	96	12

Table S3. Pan-cancer proteome-based subtypes (s1 to s11) in UALCAN database

Subtype	Go term enrichment	Pathway signatures
s1	nuclear ubiquitin ligase complex, poly-UBQ modification protein binding, nucleus	Pentose phosphate, Glycolysis/GNG, Myc, Wnt
s2	immune system process, adaptive immune response, leukocyte activation, reg. of immune system process, intrinsic component of membrane	Ras, Wnt, hypoxia
s3	cell division, cell cycle process, cytosolic large ribosomal subunit, chromosome segregation	YAP1,Myc,Wnt
s4	mitochondrion, fatty acid oxidation, tricarboxylic acid cycle, ion transmembrane transport, oxidative phosphorylation	FA metabolism, Glycolysis/GNG, TCA cycle, OX-PHOS, Ras
s5	reg. of immune system process, extracellular region, extracellular matrix,vesicle	EMT, hypoxia, NRF2/KEAP1, Notch
s6	extracellular region, extracellular matrix, cell adhesion, focal adhesion, cytoskeleton, cell junction	EMT, hypoxia, NRF2/KEAP1
s7	cell junction, axon guidance, frizzled binding, locomotion	EMT, Notch
s8	immune system process, adaptive immune response, leukocyte activation,extracellular region, neutrophil activation, exocytosis, humoral immune response	Glycolysis/GNG, Pentose phosphate, Ras, EMT, hypoxia
s9	fatty acid oxidation, intrinsic component of membrane, glycosylation	NRF2/KEAP1, Notch
s10	RNA splicing, nucleus, DNA repair, cell division, cell cycle process	YAP1, Myc,Wnt
s11	cell junction, synapse, dendrite, axon	Glycolysis/GNG, TCA cycle, OX-PHOS, NRF2/KEAP1

Table S4. Pan-cancer proteinomic analysis of SLC2A1

Comparison	Statistical significance
**S1-vs-S2**	**9.30E-08**
**S1-vs-S3**	**1.14E-12**
**S1-vs-S4**	**6.06E-05**
**S1-vs-S5**	**6.83E-05**
**S1-vs-S6**	**1.50E-02**
**S1-vs-S7**	**1.96E-05**
**S1-vs-S8**	**2.79E-18**
**S1-vs-S9**	**6.54E-07**
**S1-vs-S10**	**9.53E-08**
**S1-vs-S11**	**4.29E-03**
**S2-vs-S3**	**3.43E-04**
**S2-vs-S4**	**4.09E-03**
S2-vs-S5	2.41E-01
**S2-vs-S6**	**8.53E-06**
**S2-vs-S7**	**3.52E-02**
**S2-vs-S8**	**5.44E-13**
S2-vs-S9	7.79E-01
S2-vs-S10	7.67E-01
**S2-vs-S11**	**1.31E-04**
**S3-vs-S4**	**1.31E-08**
**S3-vs-S5**	**4.99E-05**
**S3-vs-S6**	**2.61E-11**
**S3-vs-S7**	**2.44E-07**
**S3-vs-S8**	**2.89E-02**
**S3-vs-S9**	**3.02E-04**
**S3-vs-S10**	**6.83E-05**
**S3-vs-S11**	**4.30E-10**
S4-vs-S5	3.68E-01
**S4-vs-S6**	**2.02E-02**
S4-vs-S7	4.86E-01
**S4-vs-S8**	**5.29E-26**
**S4-vs-S9**	**2.48E-02**
**S4-vs-S10**	**2.92E-03**
S4-vs-S11	1.10E-01
**S5-vs-S6**	**1.63E-02**
S5-vs-S7	6.80E-01
**S5-vs-S8**	**1.35E-11**
S5-vs-S9	3.81E-01
S5-vs-S10	3.07E-01
S5-vs-S11	5.52E-02

**S6-vs-S7**	**6.07E-03**
**S6-vs-S8**	**3.77E-28**
**S6-vs-S9**	**1.56E-04**
**S6-vs-S10**	**3.12E-06**
S6-vs-S11	5.38E-01
**S7-vs-S8**	**1.67E-21**
S7-vs-S9	1.08E-01
**S7-vs-S10**	**3.93E-02**
**S7-vs-S11**	**3.84E-02**
**S8-vs-S9**	**1.52E-11**
**S8-vs-S10**	**2.13E-16**
**S8-vs-S11**	**2.90E-24**
S9-vs-S10	9.68E-01
**S9-vs-S11**	**1.25E-03**
**S10-vs-S11**	**7.84E-05**

Table S5. GO/KEGG analysis of SLC2A1-related DEGs.

Table S6. GSEA of SLC2A1 based on HALLMARK gene sets.

Table S7. GSEA of SLC2A1 based on REACTOME gene sets.

Tables S5.–S7. are provided separately, attached as Excel files.
